# Extensive Bilateral Extraluminal Perivascular Pulmonary Haemorrhage Associated with Stanford Type A Aortic Dissection

**DOI:** 10.1155/2011/681643

**Published:** 2011-08-11

**Authors:** Angeline Reid, Peter Stride, Jonathan Hunter, Katerina Liew, T. Wood, Mostafa Seleem

**Affiliations:** ^1^Redcliffe Hospital, Locked Bag 1, Redcliffe, Queensland 4020, Australia; ^2^University of Queensland School of Medicine, Redcliffe Hospital, Locked Bag 1, Redcliffe, Queensland 4020, Australia

## Abstract

We present the case of an 80-year old man with a Stanford Type A dissecting thoracic aortic aneurysm plus the unusual CT finding of extramural haemorrhage along the pulmonary vessels. The clinical and radiological picture has an extremely high mortality

## 1. Case Report

An 80-year-old man presented to the emergency department having collapsed suddenly with leg weakness. On admission, he was hypotensive with peripheral cyanosis and hypoperfusion. Examination revealed a flaccid paraplegia with absent leg reflexes, yet palpable femoral pulses. He had a history of hypertension, and investigations twelve months prior to presentation demonstrated a distal abdominal aortic aneurysm measuring 2.5 cm in diameter and 3 cm in length.

**Figure 1:**
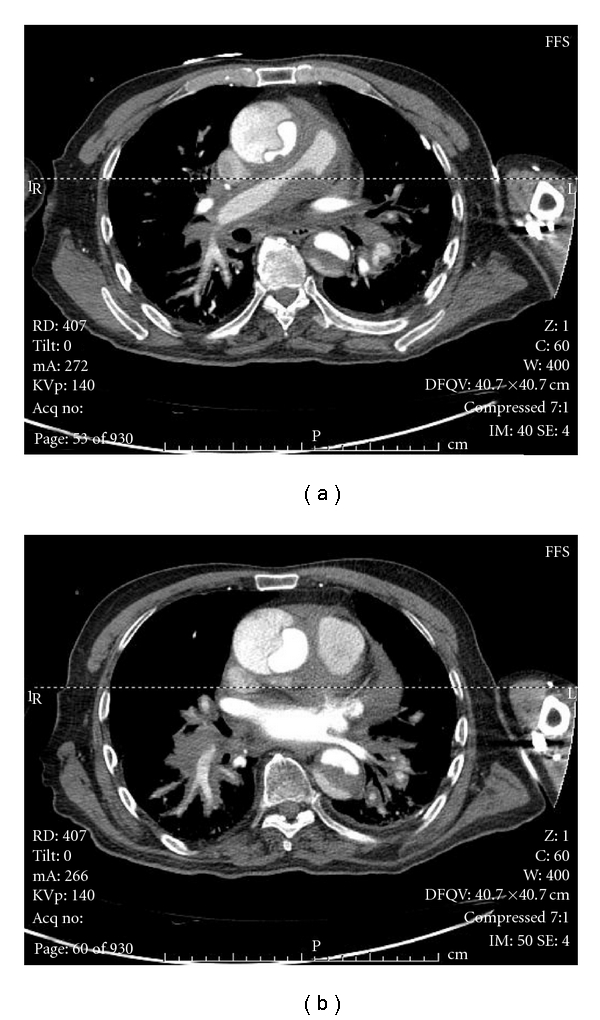
CT scans demonstrate an aortic aneurysm, showing contrast within the true lumen, a pericardial effusion, and haemorrhage extending down the pulmonary arteries and veins to subsegmental branches.

Emergency CT (Figure 1) demonstrated a dissecting aneurysm of the thoracic ascending aorta (Stanford Type A) plus a small pericardial effusion and the unusual picture of bilateral extraluminal perivascular pulmonary haemorrhage with extension down the segmental and subsegmental pulmonary arteries and veins. The dissection involved the right renal artery inferiorly but did not extend below this. His case was discussed with the nearest cardiac surgical unit, that felt that no feasible intervention could be offered, and his prognosis was poor with exceptionally high mortality risk. Following discussion with the family, he was provided with comfort measures, and he died five hours after presentation. 

## 2. Discussion

Intramural dissection back down the aorta into the pericardium causing tamponade can occur with Stanford Type A aortic dissections and indicates an increased risk of mortality. Gilon et al. [1] reported cardiac tamponade in 126 (18.7%) of a series of 674 patients from the International Registry of Acute Aortic Dissection. the comparative inhospital mortality rate was 54%, of those patients with cardiac tamponade, compared with 24.6% in those patients without tamponade. Ninety-two percent of those patients with tamponade who were managed conservatively died. 

The CT was initially reported as showing dissection of both the aorta and pulmonary artery. However, this is exceedingly rare, usually rapidly fatal and occurs most frequently in association with pulmonary hypertension [2]. Pulmonary artery dissections are diagnosed during life in only 14% of cases and are most commonly diagnosed at autopsy. They usually occur at the site of an aneurysm or dilatation and occasionally following pulmonary artery catheterization or surgery. They predominantly cause haemorrhage at the pulmonic trunk and rupture into the pericardial sac, with cardiac tamponade as the principle mechanism of death [3]. 

However, a radiological review noted that the pulmonary veins were also involved, thus excluding dissection, which does not occur in the low pressure venous system. The revised report described the still unusual image of extra-luminal perivascular extravasation of blood along both the pulmonary arteries and veins. The ascending aorta and pulmonary trunk have a common adventitial plane in the bronchovascular sheath at the root of the great vessels and extravasated blood from the aorta can track along the pulmonary vessels [4].

Sueyoshi et al. [4] noted haemorrhage along the pulmonary artery in twenty-one cases (9.1%) in a series of 232 patients with a Stanford Type A aortic dissection. The four patients with external compression and resulting stenosis of the main pulmonary trunk from extravasated blood extending between the adventitia and media of the pulmonary trunk all died. Seven of the twenty-one patients were managed nonoperatively with three deaths. Five of the six patients (85%) with haemorrhage extending past the interlobular septa into the alveoli died [4]. 

## 3. Conclusion

A thoracic CT scan showing a Stanford Type A dissecting aneurysm with a haemorrhagic pericardial effusion and intrapulmonary extramural perivascular extravasation of blood into the alveoli implies a very poor prognosis.
